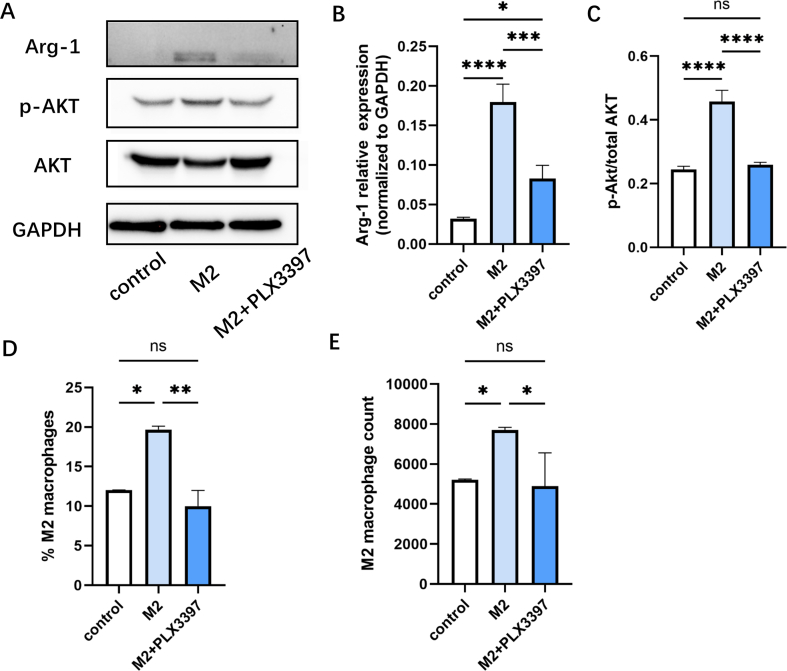# Colony-stimulating factor-1 receptor inhibition combined with paclitaxel exerts effective antitumor effects in the treatment of ovarian cancer

**DOI:** 10.1016/j.gendis.2023.04.023

**Published:** 2023-06-22

**Authors:** Meijia Yu, Yiming Wu, Qingfang Li, Weiqi Hong, Yang Yang, Xiaoyi Hu, Yanfei Yang, Tianqi Lu, Xia Zhao, Xiawei Wei

**Affiliations:** a Laboratory of Aging Research and Cancer Drug Target, State Key Laboratory of Biotherapy and Cancer Center, National Clinical Research Center for Geriatrics, West China Hospital, Sichuan University, Chengdu, Sichuan 610041, China; b Department of Gynecology and Obstetrics, Development and Related Disease of Women and Children Key Laboratory of Sichuan Province, Key Laboratory of Birth Defects and Related Diseases of Women and Children, Ministry of Education, West China Second Hospital, Sichuan University, Chengdu, Sichuan 610041, China; c Department of Obstetrics and Gynecology, The First Affiliated Hospital, Army Medical University, Chongqing 400038, China

**Keywords:** CSF-1R, Ovarian cancer, Paclitaxel, PLX3397, Targeted therapy, Tumor-associated macrophages

## Abstract

Ovarian cancer is the tumor with the highest mortality among gynecological malignancies. Studies have confirmed that paclitaxel chemoresistance is associated with increased infiltration of tumor-associated macrophages (TAMs) in the microenvironment. Colony-stimulating factor 1 (CSF-1) receptor (CSF-1R) plays a key role in regulating the number and differentiation of macrophages in certain solid tumors. There are few reports on the effects of targeted inhibition of CSF-1R in combination with chemotherapy on ovarian cancer and the tumor microenvironment. Here, we explored the antitumor efficacy and possible mechanisms of the CSF − 1R inhibitor pexidartinib (PLX3397) when combined with the first-line chemotherapeutic agent paclitaxel in the treatment of ovarian cancer. We found that CSF-1R is highly expressed in ovarian cancer cells and correlates with poor prognosis. Treatment by PLX3397 in combination with paclitaxel significantly inhibited the growth of ovarian cancer both *in vitro* and *in vivo*. Blockade of CSF-1R altered the macrophage phenotype and reprogrammed the immunosuppressive cell population in the tumor microenvironment.

## Introduction

Ovarian cancer, as a malignant tumor, has the highest mortality rate among gynecologic malignancies.^1^ Because the onset of ovarian cancer is insidious and there is no effective early diagnosis method, most patients are in the advanced stage of the disease when detected. Patients treated with initial chemotherapy have first remission rates of up to 80%, but 70% of them will experience tumor recurrence within 5 years and gradually develop chemoresistance with increasing chemotherapy,^2^ so how to enhance the efficacy of chemotherapeutic agents has become an urgent clinical problem. Tumor cells, cytokines, chemokines, and mesenchymal cells together constitute the tumor microenvironment (TME), in which the interaction between mesenchymal cells and tumor cells runs through the whole process of tumor development, such as tumor cell proliferation, angiogenesis, metastasis, invasion, and influence on circulating tumor cells.^3^ Immunosuppression of the TME is the main cause of tumor resistance and a high recurrence rate after treatment.^4^ Macrophages are plastic, as they can acquire different functional phenotypes depending on their environment. Most studies have demonstrated that M1 macrophages possess both strong microbicidal and antitumor effects, while M2 macrophages promote tumor development.^5^ In the TME, recruited macrophages are “reeducated” into tumor-associated macrophages (TAMs) with M2-like macrophage characteristics through various molecular regulatory mechanisms. TAMs are highly expressed in a variety of tumor tissues and show a direct relationship with tumor prognosis. In breast, prostate, endometrial, bladder, and esophageal epithelial and squamous cell carcinomas, there is a significant positive correlation between the level of TAM infiltration and poor prognosis.^6^ Studies have indicated that infiltration of TAMs in the TME may increase after chemotherapy, which diminishes the chemotherapy drug-induced killing effect of tumor cells and creates chemoresistance.^7^

Macrophage colony-stimulating factor 1 (CSF-1) receptor (CSF-1R), encoded by the *c-Fms* proto-oncogene, is a type of receptor tyrosine kinase^8^ and plays an important role in the regulation of macrophage proliferation and differentiation, as well as a key factor in macrophage phenotypic alteration.^9^ CSF-1R consists of five immunoglobulin structural domains, a transmembrane region, and an intracellular region. CSF-1 binding to the extracellular region of CSF-1R can induce homodimerization of intracellular tyrosine residues, which then activates receptor signaling of CSF-1R and CSF-1R tyrosine kinase-mediated autophosphorylation. The CSF-1R signaling pathway regulates macrophage production, survival, and function through the activation of downstream intracellular signaling pathways,^10^^,^^11^ including the phosphatidylinositol 3-kinase/phosphorylated AKT (PI3K/PAKT) and phospholipase C pathways, which regulate macrophage survival, and the mitogen-activated protein kinase and PI3K pathways, which regulate macrophage proliferation.^12^ High expression of CSF-1R in tumor tissues or serum of patients with breast cancer, ovarian cancer, Hodgkin's lymphoma, *etc.*, was found to be closely associated with poor prognosis.^13^, ^14^, ^15^, ^16^ It has been postulated that CSF-1 secreted by tumor cells binds to CSF-1R to recruit macrophages into the TME and differentiate into TAMs, which are involved in tumor cell growth, metastasis, angiogenesis, and immunosuppression.^17^

Pexidartinib (PLX3397) is a small molecule tyrosine kinase inhibitor that can act on CSF-1R, Kit (c-Kit), and FLT3 targets. *In vitro* studies revealed that PLX3397 inhibited tumor cell proliferation in M-NFS-60, Bac1.2F5, and M-07e cells. Meanwhile, the autophosphorylation of the CSF-1R signaling pathway is effectively inhibited by PLX3397, resulting in the inhibition of the downstream signaling molecules PI3K, AKT, ERK, and RAF, which affect tumor cell function.^18^^,^^19^ *In vivo* studies found that pexidartinib/PTX treatment led to a remarkable reduction in CD31^+^ vascular density within mammary tumors and induced apoptosis and necrosis of tumor cells in the MMTV-PyMT mouse model of breast cancer.^7^ To date, preclinical studies on the antitumor effects of PLX3397 have been conducted in hepatocellular carcinoma, cervical cancer, and melanoma.^20^, ^21^, ^22^, ^23^ In contrast, the antitumor effects and mechanism of PLX3397 in ovarian cancer cells remain to be elucidated in preclinical studies and are still in the exploratory stage,^21^^,^^24^ while basic studies of PLX3397 in combination with paclitaxel in ovarian cancer have not been reported. Since the literature and previous studies in our laboratory reported elevated TAMs triggered by paclitaxel chemotherapy, this research aimed to investigate whether the combination of PLX3397 and paclitaxel could enhance the antitumor effect on ovarian cancer and to elucidate the related mechanism. The relationship between CSF-1R expression and the prognosis of ovarian cancer patients was investigated by analyzing the expression of CSF-1R in ovarian cancer cells via a tissue microarray of primary ovarian cancer. The inhibitory effects of PLX3397 on the proliferation and cell migration of ovarian cancer cells were probed. Using paclitaxel as a model drug in combination with a CSF-1R inhibitor, we explored the antitumor effects of this regimen and the drug safety in the treatment of the ovarian cancer ID8 animal model. The impact of the combination of CSF-1R inhibitors and chemotherapeutic agents on the TME was characterized, providing more potential targets for the clinical treatment of ovarian cancer.

## Materials and methods

### Patient cohorts and tissue specimens

The tumor specimens and clinical data of patients involved in this research were provided by Shanghai Xinchao Biotechnology Co., Ltd. and were ethically approved. Case inclusion criteria: 111 cases with a pathological diagnosis of primary ovarian cancer. All cases were operated on between February 12, 2009 and February 25, 2013. Follow-up started on the day of operation, postoperatively by telephone follow-up, and ended in March 2018 or at death, with no lost cases. Relevant basic clinicopathological data of the patients were also collected and recorded. The intensity and positivity of immunohistochemical staining of tumor tissues were independently judged by two experienced pathologists and scored according to the following criteria. Scoring of staining intensity: 0 (negative), 1 (1+), 2 (2+), 3 (3+); scoring of staining positivity: 0 (negative), 1 (1%–25%), 2 (26%–50%), 3 (51%–75%), 4 (76%–100%). Tissues were grouped by the product of staining intensity score and staining positivity score as the total score; 0 (negative), 2–3 (mild), 4–8 (moderate), and 9–12 (strongly positive). Negative and mild are low expressions, and moderate and strong positive are high expressions.

### Cell lines and animals

The mouse plasmacytoid ovarian cancer cell line ID8 and human-derived ovarian cancer cell line A2780 were provided by the State Key Laboratory of Biotherapy, Sichuan University. The medium used for culturing the above cells was DMEM (Dulbecco's Modified Eagle Medium) complete medium and 1640 medium containing 10% or 20% fetal bovine serum, 100 U/mL penicillin, and 100 μg/mL streptomycin. Cells were cultured at 37 °C in a cell culture incubator containing 5% CO_2_.

The mice for this research were female SPF-grade C57BL/6, aged 6–8 weeks, weighing approximately 18–20 g. They were purchased from Beijing Huafukang Laboratory Animal Technology Co., Ltd. and housed in the Animal Center of State Key Laboratory of Biotherapy, High-Tech Zone, Sichuan University. All animal experiments were reviewed and approved by the State Key Laboratory of Biotherapy Animal Care and Use Committee of Sichuan University, Sichuan, China.

### Reagents and antibodies

Paclitaxel was purchased from Shangdong Qilu Company. PLX3397 was provided by WuXi AppTec. For *in vitro* experiments, PLX3397 was dissolved in DMSO solution, prepared as the stock solution at a concentration of 20 mM, and stored at −80 °C for backup. When used, the solution was diluted to the appropriate concentration according to the experimental needs. For *in vivo* experiments, 0.5% methylcellulose (MC) was used as a blank solvent control. PLX3397 was dissolved in 0.5% MC, prepared as a 5 mg/mL dosing preparation, and stored in a refrigerator at 2–8 °C. Paclitaxel was prepared in sterile normal saline to 1 mg/mL. Annexin V-FITC apoptosis detection kits were purchased from BD, U.S.A. CCK-8 cytotoxicity kits were purchased from MCE, China. Anti-MCSF receptor antibody (ab183316) and anti-CSF-1R antibody (ab254357) were purchased from Abcam, U.K. Anti phospho-CSF-1R (Tyr723) antibody (3155), anti AKT antibody (4691), and anti phospho-Akt (Ser473) antibody (4060) were purchased from Cell Signaling Technology. EnVision™ FLEX+, Mouse, High PH (Link) was purchased from Dako, China. The required flow indicator antibodies, anti-CD45, anti-CD11b, anti-F4/80, anti-CD206, anti-Ly6G, anti-Ly6C, anti-Gr-1, anti-CD3, anti-CD4, anti-CD8, and anti-CD69, were purchased from Abcam. The required immunofluorescent primary antibody reagents F4/80 (GB11027, 1:200), Ki67 (GB13428, 1:300), and CD31 (GB13428, 1:500) were purchased from ServiceBio, China, and VEGF (Ab52917, 1:100) was purchased from Abcam. The required immunohistochemical antibodies, anti-CD206 (1:100) and CSF-1R (1:10), were purchased from BD.

### Cell proliferation assay

Cells at the logarithmic growth stage were collected and made into a single-cell suspension, and the number of cells was counted using a Countstar automated cell counting plate. The cell density was calculated based on the number of cells and the duration of the experiment, and the cell suspension was seeded into 96-well plates with 100 μL medium per well and incubated overnight. Then, 100 μL medium containing the appropriate drug concentration was added to each well and incubated for 24, 48, and 72 h. Finally, cell proliferation was assayed using the Cell Counting Kit-8 (CCK-8) according to the manufacturer's instructions.

### Cell cycle PI staining assay

Cells were seeded in 6-well plates and treated with drugs for 24 h using the method described above. The collected cells were then rinsed with PBS and fixed overnight in 70% ethanol at 4 °C. Finally, the samples with RNase added were stained with PI (50 mg/mL). Red fluorescence and scattering at 488 nm were detected by flow cytometry for analyzing the cell cycle.

### Cell apoptosis assay

Cells were seeded in 6-well plates and treated with drugs for 24 h using the method described above. Then, the cells were harvested and washed twice with ice-cold PBS. Apoptosis was detected according to the instructions of a BD Biosciences Annexin-V/PI double-stained apoptosis kit.

### Colony formation assays

Cells in the logarithmic growth phase were collected and made into single-cell suspensions. A total of 50 to 100 cells were seeded per well in 6-well plates. The drug was added to the corresponding group of cells and then cultured in an incubator. When the control showed visible colony formation, the culture was terminated; the culture medium was discarded and the cells were washed twice with precooled PBS. The cells were fixed with prechilled 4% paraformaldehyde for 10–15 min and then stained with crystal violet staining solution for 10–15 min. After natural drying, colony formation was observed under an inverted microscope and photographed.

### Wound-healing assay and transwell migration assay

Tumor cells were seeded on 6-well plates and cultured to 90%, and the traumatic surface was uniformly scratched with the tip of a sterile pipette. The culture was continued with the addition of a medium containing the appropriate drug concentration. Cell motility was assessed by measuring the migration of cells to scratch wounds. Wound healing was monitored by measuring the scratch width at 0 h, 24 h, and 48 h, and the percentage of cell migration area was calculated and counted for each time point relative to 0 h.

Starvation-treated cells (containing different drug concentrations) were added to the top chambers of the 24-well invasion chambers. After culture in a constant temperature incubator for 24 h, the cells were fixed with 4% paraformaldehyde for 10–15 min and stained with 0.5% crystal violet for 10–15 min, and the images were recorded by microscopy after natural air-drying.

### *In vivo* experiments

Mouse ovarian cancer ID8 cells were injected intraperitoneally into female C57BL/6 mice (5–10 × 10^6^ cells/mouse) to establish an ID8 intraperitoneal metastasis model. After the establishment of the animal model, the tumor-bearing mice were randomly divided into the vehicle group, CSF-1R inhibitor group, paclitaxel group, and drug combination group (5–7 mice per group). The CSF-1R inhibitor PLX3397 was 5 mg/mL, and paclitaxel was 1 mg/mL. The administration was started on the tenth day after a tumor challenge. The vehicle group was treated with an equal volume of 0.5% MC solvent by gavage; the CSF-1R inhibitor group was given PLX3397 at 45 mg/kg by gavage for 21 days; the paclitaxel group was given paclitaxel at 10 mg/kg once by intraperitoneal administration; and the drug combination group was given both the above-used agents at the same dosage. The body weight of the mice was recorded every three days, and the curve of body weight change and tumor load change was plotted. The health status of the mice was observed daily during drug administration. At the end of drug administration, blood from the eyes of mice and ascites of the mice were taken for blood biochemical indexes and microenvironment indexes, respectively. Tumor nodules were weighed and photographed, and important organs were subsequently paraffin-embedded, sectioned, and HE-stained.

### Immunohistochemistry (IHC)

Fresh tumor nodule sections embedded in 4% methanol-free formaldehyde were stained with the appropriate antibodies to detect specific molecules' expression. Tumor sections were immunohistochemically stained according to the manufacturer's instructions. Images of IHC were obtained with light microscopy.

### Flow cytometry analysis

Ascites were collected after the execution of the mice. If there are no obvious ascites, saline or PBS can be used to flush the peritoneal cavity, and then the peritoneal lavage fluid can be taken. The obtained ascites were centrifuged to make a cell suspension, and 1 μL of antibody was added to every 100 μL of suspension. The expression of macrophages (CD45^+^ F4/80^+^ CD206^+^ CD11b^+^ MHCII^+^), neutrophils (CD11b^+^ Ly6G^+^), monocytes (CD11b^+^ Ly6C^+^), and T cells (CD4^+^ T cells, CD8^+^ T cells) in the tumor immune microenvironment was detected using flow cytometry. Data were acquired using the Novocyte system and analyzed using the appropriate Novocyte software.

### Assessment of safety and toxicity

To evaluate the toxicity of PLX3397 combined with paclitaxel *in vivo*, mouse eye blood was taken, and the serum was isolated and then analyzed biochemically with a fully automated biochemical analyzer.

### Statistical analysis

GraphPad Prism 8.0 software was used for statistical analysis of the results. The experimental results were expressed as mean ± standard deviation (SD). Comparisons between two groups were analyzed by Student's *t*-test, and comparisons between multiple groups were analyzed by ANOVA. Differences were considered statistically significant when *P* < 0.05.

## Results

### CSF-1R is highly expressed in ovarian cancer cells and correlates with poor prognosis

**Figure 1:**
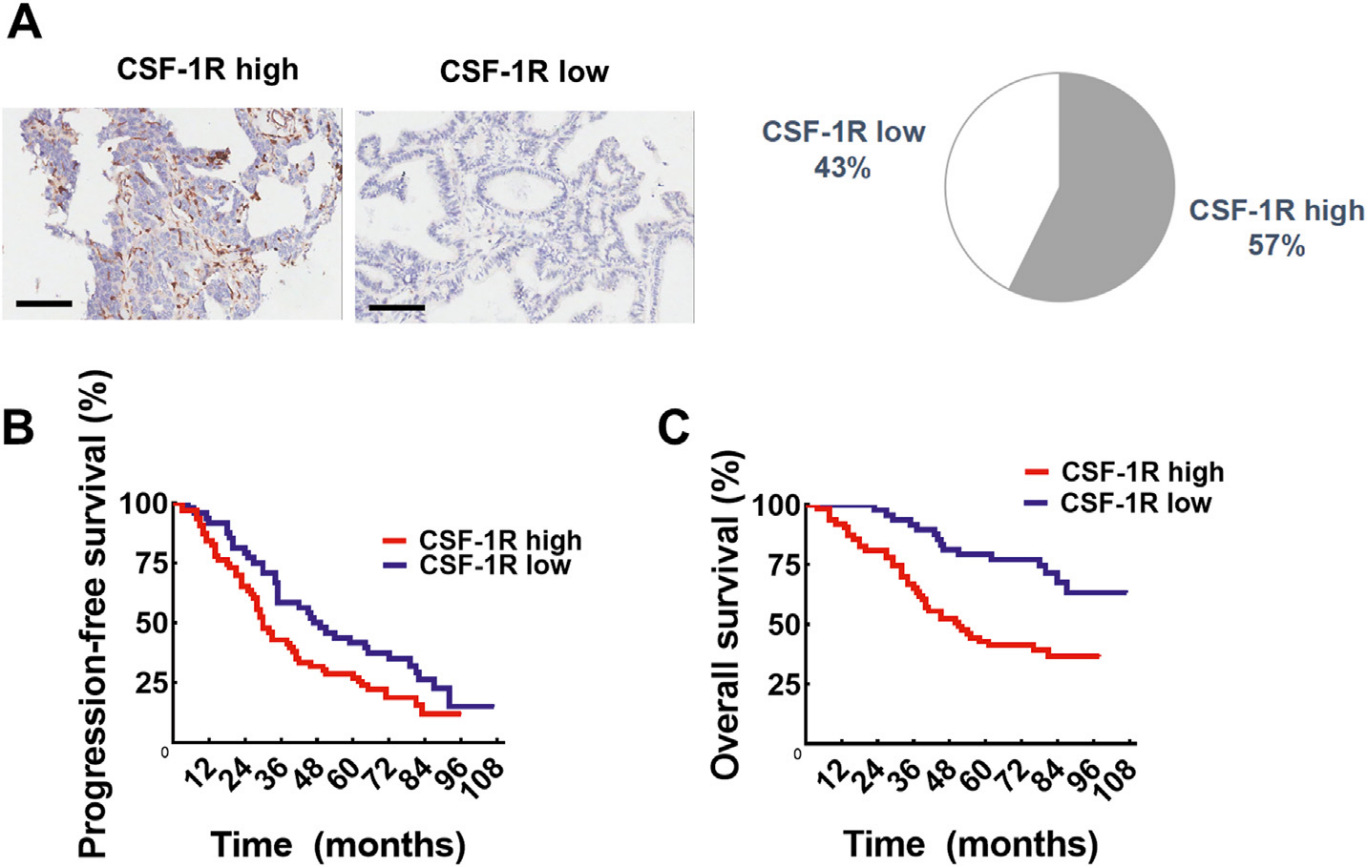
Highly expressed CSF-1R is associated with poor prognosis in ovarian cancer patients. **(A)** IHC images with labeled high/low CSF-1R were representative regions of CSF-1R expression in ovarian cancer microarray. Scale bars = 100 μm. **(B, C)** Kaplan–Meier survival curves of patients regarding different CSF-1R expression levels. Progression-free survival (B) reached a significant difference between the two groups (*P* < 0.05). Overall survival (C) reached a significant difference between the two groups (*P* < 0.001).

To investigate the importance of CSF-1R in human ovarian cancer patients, we collected tumor tissues from 111 primary ovarian cancer patients and analyzed the relationship between CSF-1R expression and ovarian cancer patient prognosis. The clinical and case characteristics of the ovarian cancer patients included in this study are summarized in Table 1. To clarify the expression of CSF-1R in the patients, we performed immunohistochemical staining of the collected tumor tissues, and two pathologists independently judged the staining intensity and positivity rate and then grouped the patients according to the scores obtained. Negative and weakly positive (score 0–3) were considered a low expression, and moderately positive and strongly positive (score 4–12) were considered a high expression, including 63 patients with CSF-1R^high^ (57.3%) and 47 patients with CSF-1R^low^ (42.7%). Representative images of the high- and low-expression groups are presented in Figure 1A. To further understand the correlation between CSF-1R expression levels and the clinicopathological characteristics of ovarian cancer patients, we statistically analyzed the CSF-1R expression levels of ovarian cancer patients with indicators related to the clinical and pathological characteristics of patients (Table 2). The results showed that patients in the high CSF-1R expression group had a higher proportion of tumor size, lymph node metastasis, and FIGO (International Federation of Obstetrics and Gynecology) stage than those in the low CSF-1R expression group. In addition, we analyzed the survival data of ovarian cancer patients in the high and low CSF-1R expression groups (Fig. 1B, C), and the results showed that the expression level of CSF-1R was significantly associated with the survival of ovarian cancer patients. These results suggest that CSF-1R is an important negative prognostic factor for human ovarian cancer.

**Table 1:** Clinical and pathological characteristics of ovarian cancer patients.

Clinical and pathological characteristics (*n* = 111)	
Age (years)	52 (42, 60)
Age (years), *n* (%)	
≤50	52 (46.8)
>50	59 (53.2)
Tumor size (cm)	12.2 (6.6, 17.1)
∗Pathological grade, *n* (%)	
Ⅰ/Ⅱ	17 (15.3)
Ⅲ	75 (67.6)
Missing	19 (17.1)
Histologic classification, *n* (%)	
Plasmacytoma	51 (45.9)
Mucinous carcinoma	21 (18.9)
Endometrioid carcinoma	9 (8.1)
Clear cell carcinoma	2 (1.8)
Mixed carcinoma	23 (20.7)
^#^Other types	5 (4.5)
TNM stage	
T stage, *n* (%)	
T1	8 (7.2)
T2	27 (24.3)
T3	76 (68.5)
N stage, *n* (%)	
N0	86 (77.5)
N1	25 (22.5)
M stage, *n* (%)	
M0	93 (83.8)
M1	18 (16.2)
FIGO stage, *n* (%)	
Stage Ⅰ	8 (7.2)
Stage Ⅱ	27 (24.3)
Stage Ⅲ	58 (52.2)
Stage Ⅳ	18 (16.2)
Recurrence, *n* (%)	
No recurrence	22 (19.8)
Recurrence	89 (80.2)

Note: ∗Histological grade was judged according to the FIGO grade criteria of the year in which the case was diagnosed. ^#^Other pathological types included malignant Brenner tumor (1 case), yolk cystic tumor (2 cases), and squamous carcinoma (2 cases). Continuous variables are expressed as median values (interquartile spacing), and categorical variables are expressed as a number of cases (percentage, %).

**Table 2:** Correlation of CSF-1R expression with clinicopathological characteristics of ovarian cancer.

Clinicopathological characteristics	CSF-1R low expression	CSF-1R high expression	*P*
Total number of cases (*n*, %)	48 (42.7)	63 (57.3)	
Age (years)	52 (44, 59)	52 (45, 61)	0.757
Size (cm)	10.3 (5.7, 14.6)	14.2 (8.9, 17.6)	0.007
Histological grade (*n*, %)			0.030
Ⅰ/Ⅱ	10 (20.8)	7 (11.1)	
Ⅲ	26 (54.2)	49 (77.8)	
Missing	12 (25.0)	7 (11.1)	
Pathological type			<0.001
Plasmacytoma	16 (33.3)	35 (55.6)	
Mucinous carcinoma	16 (33.3)	5 (7.9)	
Endometrioid carcinoma	0 (0)	9 (14.3)	
Clear cell carcinoma	0 (0)	2 (3.2)	
Mixed carcinoma	13 (27.1)	10 (15.9)	
Other types	3 (6.3)	2 (3.2)	
Lymph node metastasis			0.008
N0	43 (89.6)	43 (68.3)	
N1	5 (10.4)	20 (31.7)	
Distant metastasis (*n*, %)			0.148
M0	43 (89.6)	50 (79.4)	
M1	5 (10.4)	13 (20.6)	
FIGO staging (*n*, %)			0.016
Stage Ⅰ/Ⅱ	21 (43.8)	14 (22.2)	
Stage Ⅲ/Ⅳ	27 (56.3)	49 (77.8)	
Recurrence			
Yes	12 (25.0)	10 (15.9)	0.232
No	36 (75.0)	53 (84.1)	

### The combination of CSF-1R inhibitor with paclitaxel suppresses ovarian cancer cell proliferation *in vitro*

**Figure 2:**
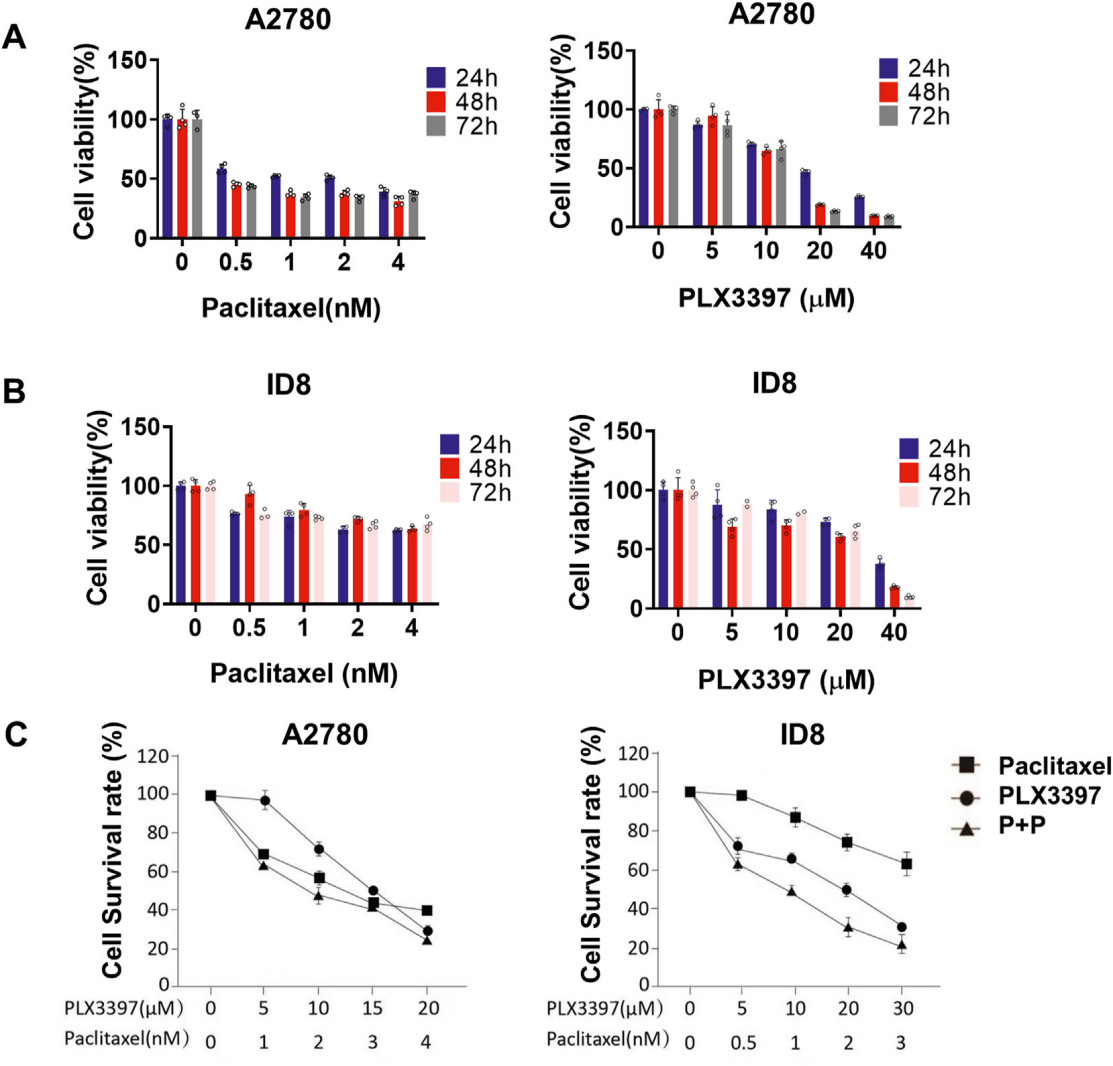
PLX3397 and the combination of PLX3397 with paclitaxel effectively suppressed ovarian cancer cell proliferation *in vitro*. **(A, B)** PLX3397 or paclitaxel inhibited the proliferation of ovarian cancer cells *in vitro*. Two different ovarian cancer cell lines A2780 (A) and ID8 (B) were cultured in 96-well plates and treated with different concentrations of PLX3397 or paclitaxel for 24 h, 48 h, and 72 h, separately. CCK8 kit was used to detect the cell viability of ovarian cancer in each group, and the results were represented by the histogram above. Data were expressed as mean ± SD. *n* = 4. **(C)** PLX3397 acted synergistically with paclitaxel in inhibiting ovarian cancer cells. Ovarian cancer cells A2780 and ID8 were treated with various concentrations of PLX3397 or paclitaxel alone or their combination for 48 h, and then the cell viability was analyzed by CCK-8 assay.

Previous papers have reported that activation of the CSF-1/CSF-1R signaling pathway promoted the autocrine cycle of CSF-1R and activated downstream signals such as AKT, leading to resistance to chemotherapeutic agents.^25^ This further supported our previous conclusion that CSF-1R is associated with poor patient prognosis. Therefore, to understand the effect of inhibiting CSF-1R on ovarian cancer cells *in vitro*, we selected the human ovarian cancer cell line A2780 and the mouse ovarian cancer cell line ID8 for *in vitro* correlation experiments. After treating the above two cell lines with different concentrations of PLX3397 (0 μM, 5 μM, 10 μM, 20 μM, 40 μM) for 24 h, 48 h, and 72 h, the number of viable cells in each group was detected by a CCK-8 kit, and the cell viability of each group was calculated after measuring the absorbance by an enzyme marker. The results showed that PLX3397, the inhibitor of CSF-1R, inhibited the proliferation of ovarian cancer cells in a time-dependent and dose-dependent manner (Fig. 2A, B). When the two drugs were combined, cell proliferation was inhibited more significantly, and the inhibitory effect on ovarian cancer cells tended to increase with increasing drug concentration (Fig. 2C).

### Multiple physiologically active hallmarks of ovarian cancer cells were attenuated *in vitro* by CSF-1R inhibitors

**Figure 3:**
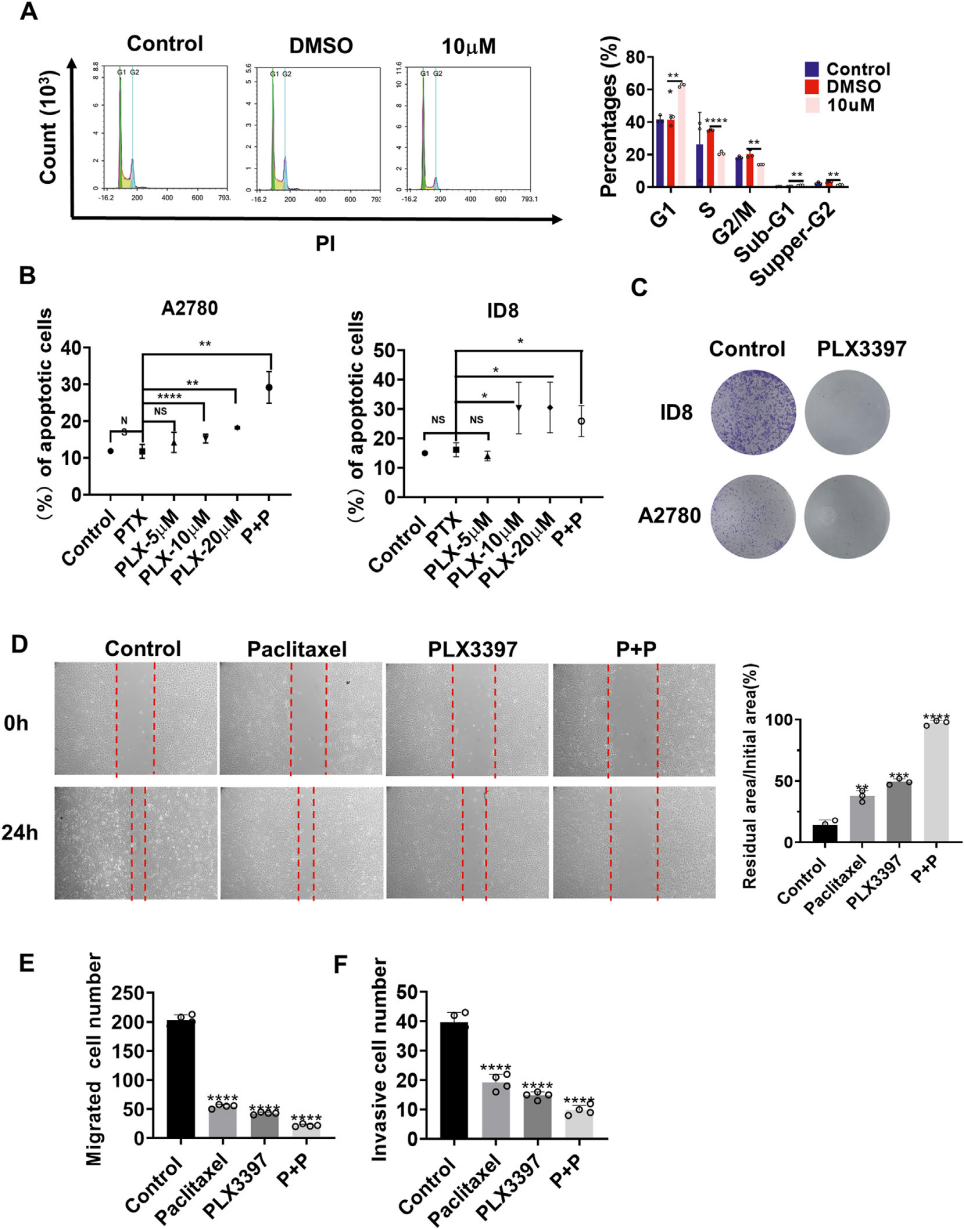
Multiple physiologically active hallmarks of ovarian cancer cells were attenuated by PLX3397 *in vitro*. **(A)** PLX3397 induced cell cycle arrest of ovarian cancer cells *in vitro*. ID8 ovarian cancer cells were treated with different concentrations of PLX3397 in 6-well culture plates for 24 h and then stained with hypotonic *p*I. The cell cycle (G1 phase, S phase, G2/M phase, Sub-G1 phase, Supper-G2 phase) of each group was detected by flow cytometry, and the percentages of each phase were statistically analyzed and compared by *t*-test. Data were presented as mean ± SD. *n* = 3. **(B)** PLX3397 induced apoptosis of ovarian cancer cells *in vitro*. PLX3397 combined with paclitaxel increased the apoptosis rate of A2780 ovarian cancer cells. A2780 and ID8 were treated with different concentrations of drugs in 6-well culture plates for 24 h. Annexin V/PI apoptotic kit staining was used to detect the proportion of apoptotic cells in each group by flow cytometry. Finally, the percentage of apoptotic cells in each group was calculated and compared. Data were presented as mean ± SD. *n* = 3. **(C)** PLX3397 inhibited the colony formation of ID8 ovarian cancer cells *in vitro*. ID8 ovarian cancer cells were treated with PLX3397 (10 μM) *in vitro*. After culture for 7 days, colony formation was observed by crystal violet staining. **(D)** PLX3397 inhibited the migration of ID8 ovarian cancer cells *in vitro*, and PLX3397 combined with paclitaxel enhanced the migration inhibition of ID8 ovarian cancer cells *in vitro*. ID8 cells were treated with different concentrations of drugs (PTX: 1 nM; PLX3397: 10 μM; P + P: 1 nM + 10 μM), and the migration ability of ID8 cells was detected by wound healing experiment. Data were presented as mean ± SD. *n* = 3. **(E, F)** PLX3397 inhibited the migration and invasion of ID8 ovarian cancer cells *in vitro*. PLX3397 combined with paclitaxel enhanced the migration and invasion of ID8 ovarian cancer cells *in vitro*. Data were presented as mean ± SD. *n* = 3. ^∗^*P* < 0.05, ^∗^^∗^*P* < 0.01, ^∗^^∗^^∗^*P* < 0.001, ^∗^^∗^^∗^^∗^*P* < 0.0001 (Student's *t*-test).

We have demonstrated the cell proliferation inhibitory effect of PLX3397 *in vitro* in the above experiments, and subsequent experiments were conducted to further understand its impacts on other physiologically active hallmarks of ovarian cancer cells *in vitro*. The results of flow cytometry after staining the cells with hypotonic PI staining solution indicated that PLX3397 induced cell cycle arrest at the G1 phase in ovarian cancer cells (Fig. 3A). In the apoptosis experiment, the cells were stained with Annexin V/PI apoptosis kit, and the proportion of apoptotic cells in each group was detected separately by flow cytometry, in which cells with early apoptosis in the Annexin V (+)/PI(−) quadrant and with late apoptosis in the Annexin V (+)/PI(+) quadrant were both considered apoptotic (Fig. S1A). The results showed that PLX3397 induced apoptosis of ovarian cancer cells *in vitro*. PLX3397 combined with paclitaxel increased the apoptosis rate of A2780 ovarian cancer cells (Fig. 3B). Additionally, PLX3397 showed the ability to inhibit colony formation in ovarian cancer cells (Fig. 3C). Finally, we conducted wound healing experiments and Transwell experiments to determine whether PLX3397 can inhibit ovarian cancer cell migration and invasion. In the wound healing experiments, the smaller the change in scratch area, the worse the migration ability of the cells, and the larger the ratio, the worse the cell migration ability. Scratch healing at 0 h and 24 h after treatment with different drug concentrations (PTX: 1 nM; PLX3397: 10 μM; P + P: 1 nM + 10 μM) is shown in Figure 3D. PLX3397 inhibited the ability of ovarian cancer cells to migrate, and the effect was more pronounced when combined with paclitaxel (Fig. 3D). In the Transwell experiments, the number of cells (ovarian cancer cells in purple) that passed through the pores to reach the lower surface of the chambers was significantly reduced with or without the addition of matrix gel to the upper surface of the chambers (Fig. S1B). The statistical results indicated that PLX3397 could inhibit the invasion and migration ability of ID8 ovarian cancer cells, and PLX3397 combined with paclitaxel could enhance its inhibitory effect (Fig. 3E, F). Finally, we also wanted to understand whether the drugs would influence CSF-1 or CSF-1R expression in tumor cells. Western blot results revealed that PTX or PLX3397 showed no effect on CSF-1 and CSF-1R expression in ID8 cells, but PTX led to an increase in the level of CSF-1R phosphorylation in the cells, whereas PLX3397 showed some degree of inhibitory tendency (Fig. S2). The data above indicated that PLX3397 inhibited multiple physiologically active characteristics of ovarian cancer cells *in vitro*.

### CSF − 1R inhibitor enhanced the antitumor efficacy of paclitaxel in ovarian cancer and improved metastasis-free survival

**Figure 4:**
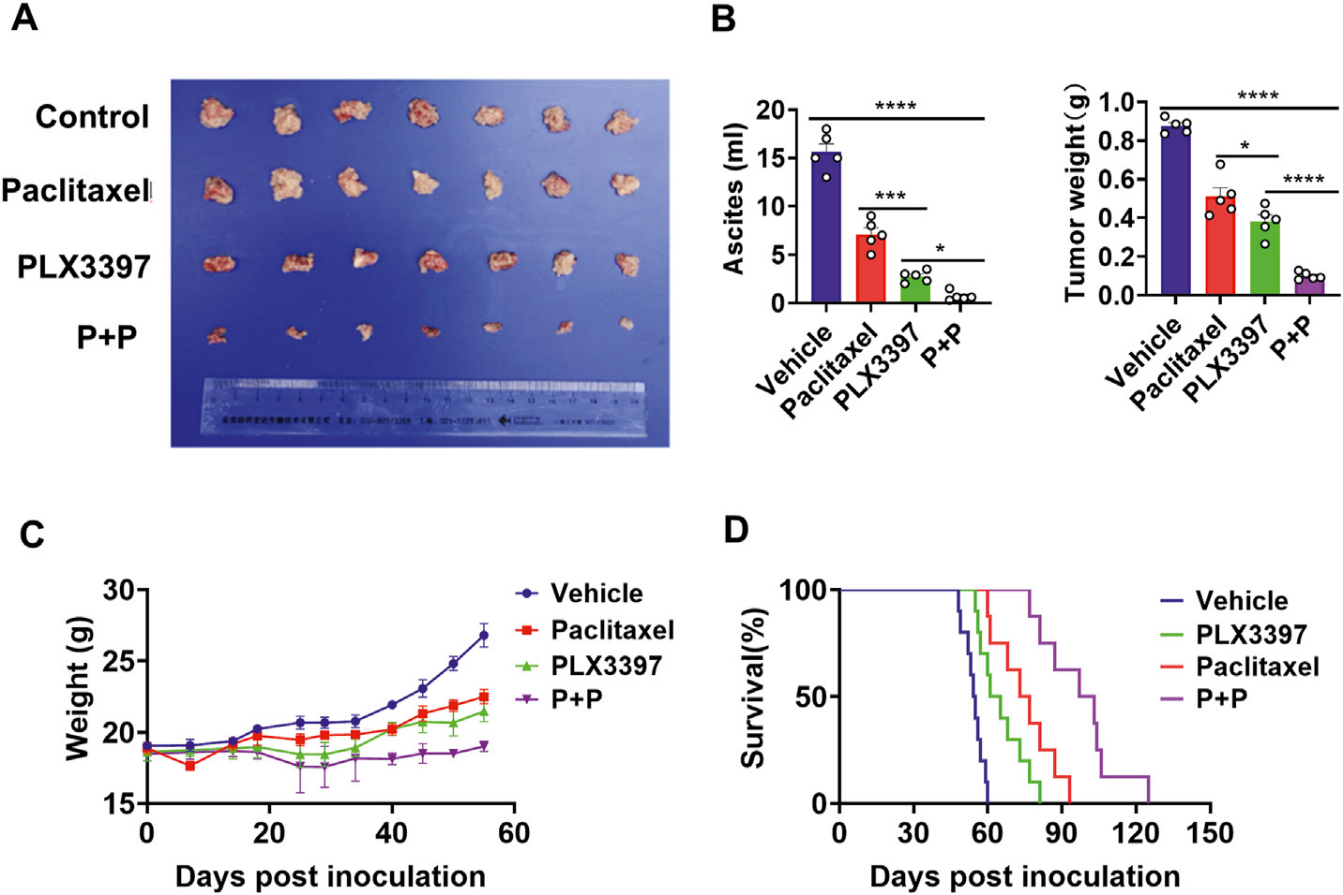
The combination of PLX3397 with paclitaxel showed promising antitumor effects and prolonged survival in tumor-bearing mice. **(A)** The gross specimens of tumor nodules in ID8 tumor-bearing mice. The tumor-bearing mice were treated with paclitaxel from the 10th day after tumor implantation, and PLX3397 was administered from the day before paclitaxel treatment. After 21 days of continuous treatment, the nodule of the abdominal tumor in the combination treatment group was significantly smaller than those in the solvent control group, paclitaxel treatment group, and PLX3397 treatment group. Data were presented as mean ± SD. *n* = 7. **(B)** The abdominal tumor nodule weight and the amount of ascites of ID8 ovarian tumor-bearing mice. Both the average weight of tumor nodules and the amount of ascites were the least in the combination group. Data were presented as mean ± SD. *n* = 5. ^∗^*P* < 0.05, ^∗^^∗^*P* < 0.01, ^∗^^∗^^∗^*P* < 0.001, ^∗^^∗^^∗^^∗^*P* < 0.0001. **(C)** Body weight growth curve of tumor-bearing mice. Data were presented as mean ± SD. *n* = 5. **(D)** PLX3397 combined with paclitaxel prolongs the survival of ID8 ovarian tumor-bearing mice. The Kaplan–Meier curve depicts the survival of ID8 tumor-bearing mice (*n* = 10/group).

To confirm the antitumor effect of CSF-1R inhibition, PLX3397, a selective CSF-1R inhibitor, combined with paclitaxel *in vivo* was used in ID8-bearing mice. The metastatic features typical of patients with ovarian malignancy in clinical practice are massive ascites formation and extensive intra-abdominal implantation, so we selected tumor nodules and ascites volume as the primary detection indicators. From the statistical results of the volume and weight of tumor nodules and the amount of ascites in the tumor-bearing mice (Fig. 4A, B), the therapeutic effect of the combined group was significantly better than that of the single drug group and the solvent control group. In addition, we recorded the changes in body weight of the mice in each group during the treatment period (Fig. 4C). 20–30 days before treatment, there was no significant change in the weight of the mice in each group. After 30 days of treatment, with the formation of ascites in tumor-bearing mice, the weight gain of mice in the solvent control group and paclitaxel group gradually exceeded that of the PLX3397 group and combination group. At the end of the observation, the average weight of mice in the combination group was the lowest. Based on the survival study of mice, we drew a survival curve of mice (Fig. 4D). From the results in the graph and our usual experiments, we found that the mice in the solvent control and PLX3397 groups died faster once ascites formed at the end of the treatment. The average survival time of mice in the solvent control group and PLX3397 group was 54.5 days and 63 days, respectively. The average survival time of mice in the paclitaxel group was 75 days and that in the combination group was significantly longer than that in the other three groups (100 days).

Moreover, we measured the expression of CD31 and vascular endothelial growth factor (VEGF) in ovarian cancer tissues of each group of mice by using immunofluorescence. As a result (Fig. S3A, B), lower expression of CD31 and VEGF was detected in the combination treatment group, suggesting that PLX3397 was able to reduce the microvessel density and inhibit neoangiogenesis in ovarian cancer tissues after paclitaxel chemotherapy. The same result was observed in the measurement of Ki67 expression (Fig. S3C), indicating that PLX3397 also inhibited cell proliferation in ovarian cancer tissues after paclitaxel chemotherapy.

### PLX3397 ameliorates the increase in M2 macrophages induced by paclitaxel treatment in the microenvironment

**Figure 5:**
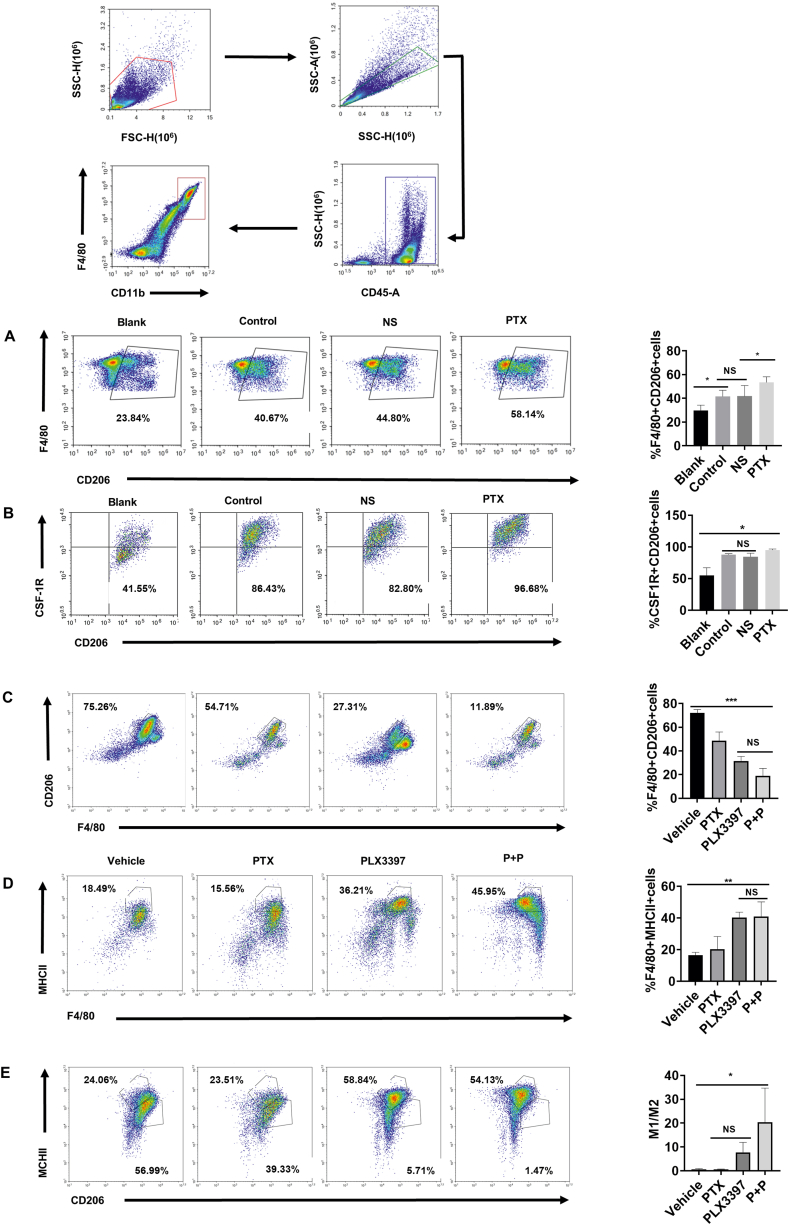
Paclitaxel treatment resulted in an increase in M2 macrophages in mice, while PLX3397 reversed this change. The first and second lines show the method of the gate. **(A, B)** Paclitaxel treatment resulted in an increase in M2 macrophages (A) and CSF-1R^+^ macrophages (B). Female C57BL/6 mice at 6–8 weeks of age were randomized into 4 groups: i) blank, ii) ID8 control, iii) ID8 + NS, and iv) ID8 + paclitaxel. Three groups were inoculated intraperitoneally with 5 × 10^6^ ID8 cells. After 10 days, and one of those groups was treated with paclitaxel (15 mg/kg, intraperitoneally). Then, the peritoneal lavage fluid was collected and cells were analyzed after 72 h. Data were expressed as mean ± SD. *n* = 4–5. ^∗^*P* < 0.05, ^∗^^∗^*P* < 0.01, ^∗^^∗^^∗^*P* < 0.001, ^∗^^∗^^∗^^∗^*P* < 0.0001. NS, not significant. **(C, D)** Decrease in M2 macrophages (C) and increase in M1 macrophages (D) caused by PLX3397 combined with paclitaxel. **(E)** Changes in the ratio of M1 macrophages to M2 macrophages. Flow cytometry was used to analyze the proportion of M2 macrophages (CD45^+^ F4/80^+^ CD206^+^) and M1 macrophages (CD45^+^ F4/80^+^ MHCII^+^) in ascites of mice in each group. Data were expressed as mean ± SD and compared by one-way ANOVA. ^∗^*P* < 0.05, ^∗^^∗^*P* < 0.01, ^∗^^∗^^∗^*P* < 0.001, ^∗^^∗^^∗^^∗^*P* < 0.0001. NS, not significant.

To investigate whether TAMs in the TME are altered after chemotherapy, animal models were utilized to simulate the changes in the peritoneal TME of human ovarian cancer. We used an early-stage abdominal tumor model of the ID8 tumor cell line, which means that the tumor load is relatively low to simulate the situation of clinical ovarian cancer patients starting chemotherapy after surgery when the patient's tumor load is low. After modeling, the peritoneal cells of mice were taken for flow cytometry analysis. The results indicated that the proportion of M2-type macrophages in the peritoneal cavity of paclitaxel-treated mice was increased compared with that of solvent control groups and tumor-untreated groups, but there was no significant difference between these two groups themselves, and both were higher than that of normal mice (Fig. 5A). This suggested that in addition to paclitaxel treatment, tumor cells alone also increased M2-type macrophages, which is similar to the results of other investigators.^26^ The percentage of M2-type macrophages in the peritoneal cavity of normal mice is approximately 20%–30%, and the results of our experiment are consistent with those of other researchers.^26^ As mentioned previously, CSF-1R is intimately associated with macrophage proliferation, differentiation, and phenotypic alterations; therefore, we tested the expression of CSF-1R-positive M2 macrophages after paclitaxel treatment in mice with peritoneal tumors. The results showed that the proportion of CSF-1R-positive M2-type macrophages in the peritoneal cells of tumor-bearing mice was increased after paclitaxel treatment (Fig. 5B). The above experiments showed that not only M2-type macrophages but also CSF-1R increased in the peritoneal cavity of the animal model of early-stage ovarian cancer after paclitaxel treatment. Next, we wanted to understand whether PLX3397 could also exert an effect on macrophages. We envisioned that PLX3397 could reduce the M2 polarization of macrophages, and thus mouse bone marrow primary macrophages were extracted and stimulated with 20 ng/mL IL-4 to polarize to the M2 type. Interestingly, we found that PLX3397 was able to inhibit the M2 polarization of macrophages *in vitro*, as evidenced by a significant decrease in Arg-1, a surface marker of M2-type macrophages (Fig. S4A, B). The results of flow cytometry also demonstrated the inhibitory effect of PLX3397 on macrophage M2 polarization from the percentage and counts of M2-type macrophages (Fig. S4D, E). Previously, it was reported that the CSF-1R inhibitor BLZ945 was able to reduce AKT phosphorylation in M1 macrophages thereby antagonizing exosome-induced M2 polarization.^27^ Therefore, we examined the phosphorylation levels of AKT in M2 macrophages treated with PLX3397 *in vitro*, and as we expected, PLX3397 significantly inhibited the phosphorylation of AKT (Fig. S4C). Based on the above experimental results, we hypothesized that PLX3397 could reduce the M2 polarization of macrophages by inhibiting AKT phosphorylation downstream of CSF-1R. To investigate whether PLX3397 could exert the same effect on macrophages *in vivo*, we analyzed the peritoneal macrophages in different treatment groups of mice. We first detected the percentage of M2-type macrophages in the combination and single drug groups, and the results are shown in Figure 5C. The proportion of M2-type macrophages was significantly lower in both the combination and PLX3397 groups than in the other two groups. Notably, in advanced ovarian cancer, the proportion of M2-type macrophages was higher in the solvent control group than in the paclitaxel-treated group, with the possible reason being that TAMs expressing high levels of CD206 in the ascites of ovarian cancer in untreated mice play an important role in promoting tumor angiogenesis,^28^ suggesting that not only do tumors need to be treated, but the negative regulation of tumor immunity generated during chemotherapy needs attention, which may be one of the mechanisms of paclitaxel chemoresistance. Additionally, the percentage of M1-type macrophages was detected (Fig. 5D). The results demonstrated that the M1-type macrophages in the combination and PLX3397 groups were clearly higher than those in the remaining two groups. Based on the above findings, we found that the enhanced antitumor effect of paclitaxel by PLX3397 could be mediated by changing the ratio of peritoneal M2-type macrophages and M1-type macrophages. Therefore, we further performed flow assays on the ratio of M1/M2 macrophages in the ascites of each group of mice, and the results are shown in Figure 5E.

**Figure 6:**
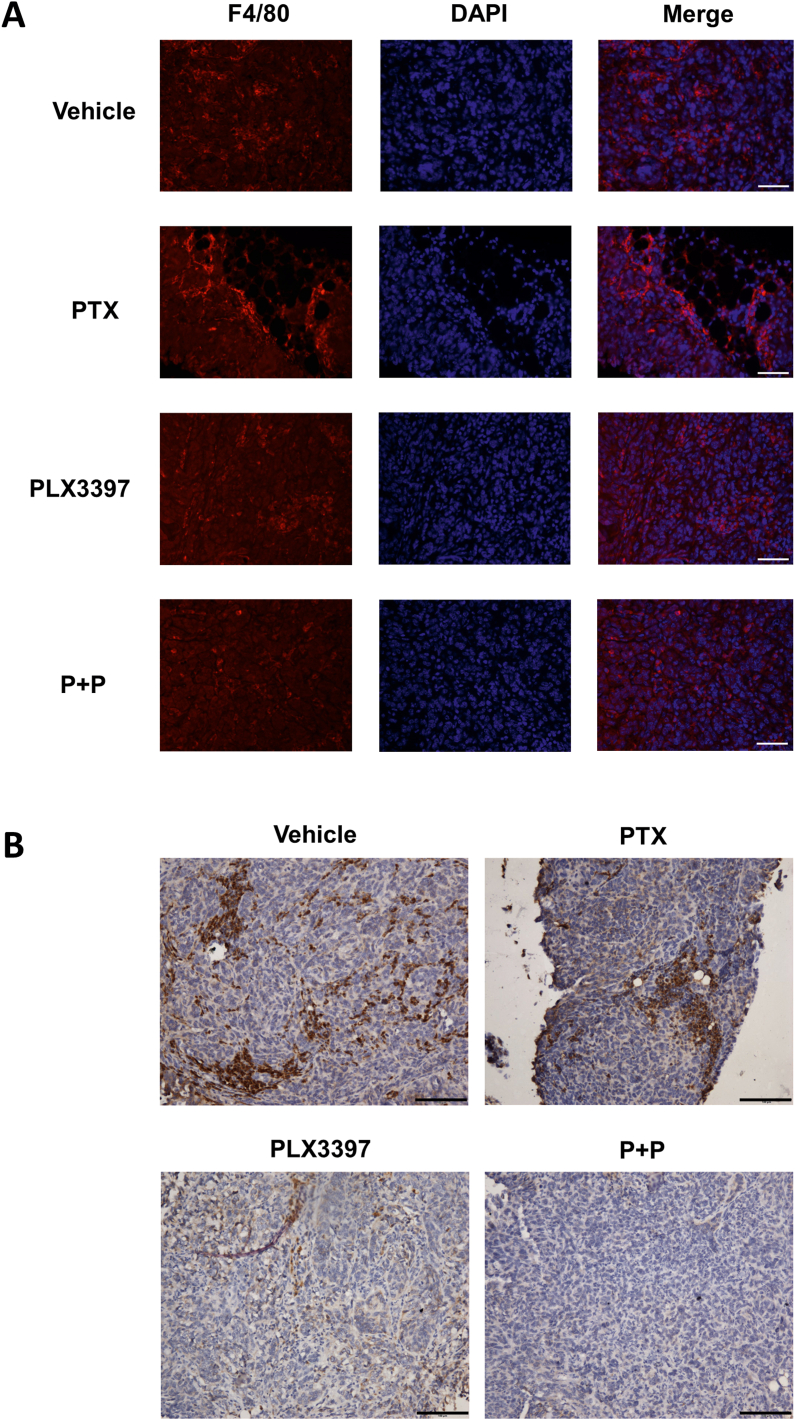
The degree of macrophage infiltration in tumor tissues after chemotherapy was reduced by PLX3397. **(A)** Effects of PLX3397 combined with paclitaxel on F4/80 expression on TAMs in tumors. Immunofluorescent detection of the macrophage marker F4/80. DAPI-stained nuclei are in blue. Scale bars = 200 μm. **(B)** The expression of CD206 was detected by immunohistochemical staining in ovarian cancer tissues of ID8 ovarian cancer/celiac tumor model treated with PLX3397 and paclitaxel. Scale bars = 100 μm.

In addition to the research on ascites, the tumor tissue of the tumor-bearing mice was also investigated. To further investigate whether PLX3397 could reduce the degree of macrophage infiltration in ovarian cancer tissues after paclitaxel chemotherapy, we measured the degree of macrophage infiltration in ovarian cancer tissues in each group of mice using immunofluorescence. We found that the degree of infiltration of F4/80-positive macrophages in the combination treatment group was lower than that in the paclitaxel treatment group alone, as shown in Figure 6A. Immunohistochemical staining results showed that the degree of M2 macrophage infiltration was lower in the combined group than in the remaining groups (Fig. 6B). In summary, we speculate that PLX3397 may achieve enhanced antitumor effects of paclitaxel by modulating changes in the macrophage phenotype in the microenvironment.

### Blockade of CSF-1R enhances the antitumor activity of paclitaxel by reprogramming immunosuppressive cell populations and altering the T cell composition in the TME

**Figure 7:**
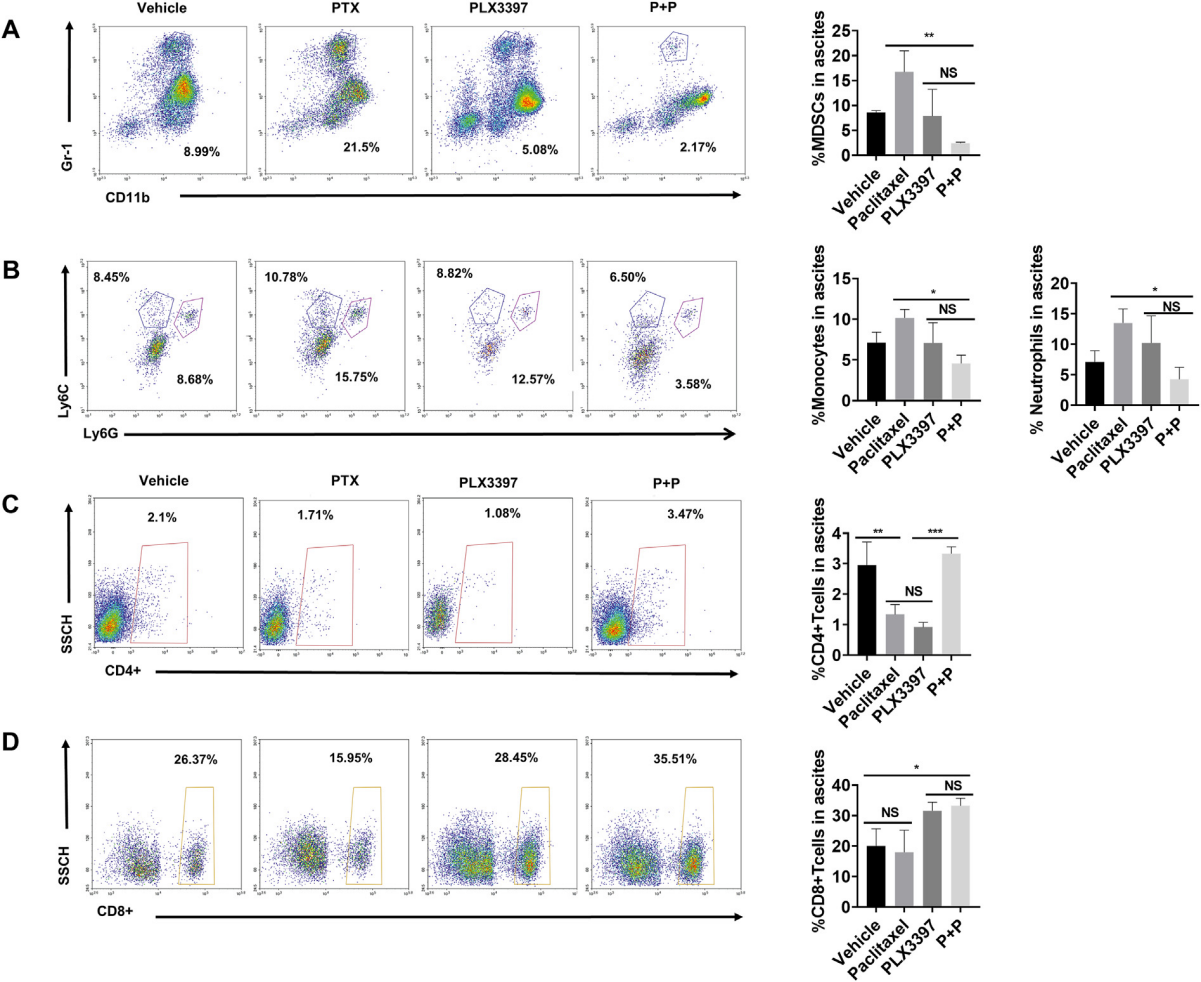
The combination of PLX3397 with paclitaxel alters the immunosuppressed tumor microenvironment. **(A**–**D)** Flow cytometry was used to analyze the proportion of MDSCs (CD45^+^ CD11b^+^ Gr-1^+^) (A), inflammatory monocytes (CD45^+^ CD11b^+^ Ly6G^+^) and neutrophils (CD45^+^ CD11b^+^ Ly6C^+^) (B), as well as CD4^+^ T cells (CD3^+^ CD4^+^) (C) and CD8^+^ T cells (CD3^+^ CD8^+^) (D) in ascites of mice in each group. Data were expressed as mean ± SD and compared by one-way ANOVA. ^∗^*P* < 0.05, ^∗^^∗^*P* < 0.01, ^∗^^∗^^∗^*P* < 0.001, ^∗^^∗^^∗^^∗^*P* < 0.0001. NS, not significant.

To gain insight into the mechanism by which PLX3397 enhances the antitumor effects of paclitaxel, we further analyzed other major cells in the TME. Myeloid-derived suppressor cells (MDSCs), inflammatory monocytes and neutrophils with negative immune regulatory or immunosuppressive effects, as well as CD4^+^ T cells and CD8^+^ T cells with positive immune regulatory effects, were detected and analyzed for changes, and the results are shown in Figure 7. The results of the flow cytometric analysis showed that the proportion of MDSCs and inflammatory monocytes and neutrophils was reduced in the combination group compared with the other three groups (Fig. 7A, B). In contrast, the proportions of CD4^+^ T cells and CD8^+^ T cells were increased (Fig. 7C, D). We found that the cells with increased proportions in the combination group were those that promoted positive immune regulation, while those with decreased proportions were usually associated with immunosuppression. The opposite result was observed in the paclitaxel monotherapy group. This implied that PLX3397 may enhance the therapeutic effect of paclitaxel by reversing the negative regulation of immunity triggered by paclitaxel and converting the immunity to the positive regulation aspect.

### PLX3397 in combination with paclitaxel shows reliable safety *in vivo*

**Figure 8:**
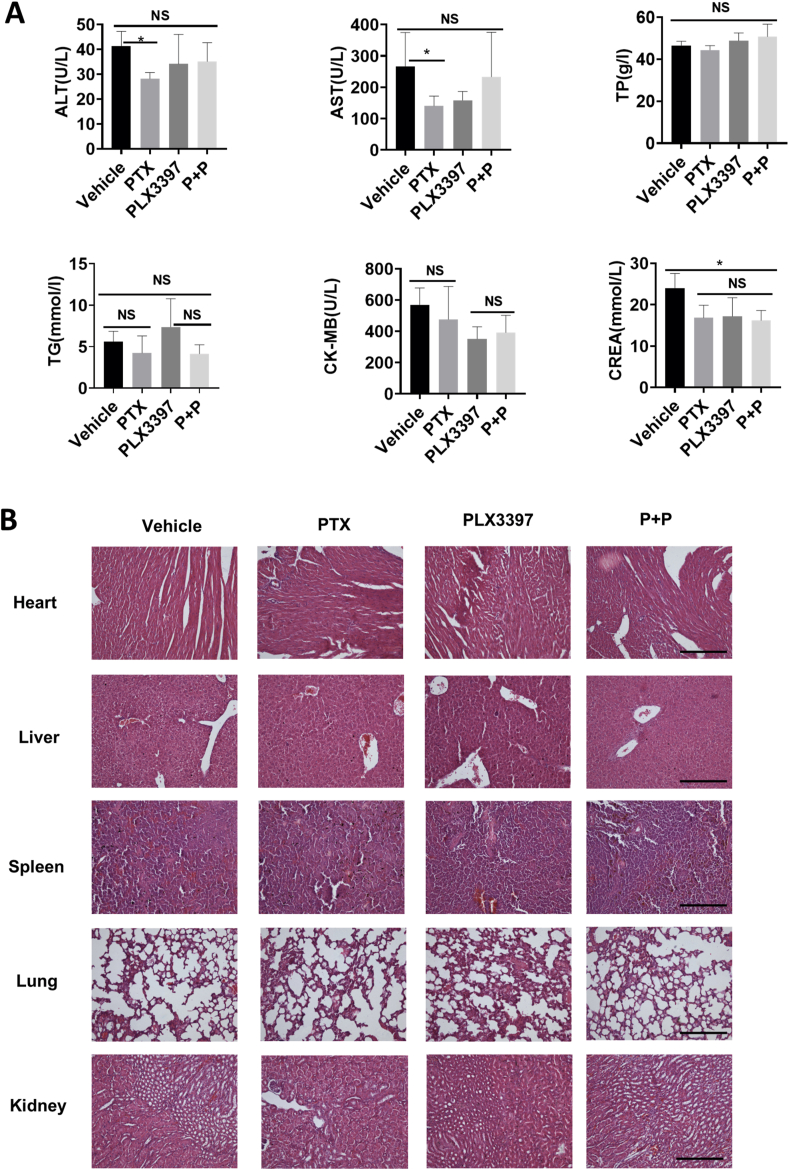
The combination of PLX3397 with paclitaxel has shown reliable safety *in vivo*. **(A)** The toxicity and safety of the drug were evaluated by biochemical analysis in mice. The main biochemical indexes of eyeball blood were detected, including AST, ALT, CK-MB, TP, TG, and Crea. Data were expressed as mean ± SD. *n* = 5. ^∗^*P* < 0.05, ^∗^^∗^*P* < 0.01, ^∗^^∗^^∗^*P* < 0.001, ^∗^^∗^^∗^^∗^*P* < 0.0001. **(B)** The pathological changes of the major organs of mice in the experimental groups were observed. The major organs (heart, liver, spleen, lung, and kidney) of ID8 abdominal tumor model mice in each group were taken for pathological sections and HE staining, and no significant pathological histological changes were found in the major organs of the mice in each group. Scale bar = 100 μm.

To test the potential side effects of the combination of PLX3397 with paclitaxel, serum biochemical analysis and hematoxylin and eosin (H&E) staining of vital organs were performed. Serum biochemical analysis revealed that alanine transaminase (ALT), aspartate aminotransferase (AST), and creatinine (Crea) were increased in the vehicle group compared with the other three groups (Fig. 8A). Considering the changes in the above three indexes, we considered that the health condition of the mice treated with the vehicle only changed later as the tumor progressed, while the drug treatment itself had no significant toxic effects on the above three test indexes in mice. The remaining three indexes (total protein (TP), triglyceride (TG), creatine kinase-MB (CK-MB)) did not show obvious differences (Fig. 8A). Meanwhile, staining of vital organs showed no obvious pathological morphological changes in the important organs (heart, liver, spleen, lung, and kidney) in mice (Fig. 8B). The above results indicated that PLX3397 possesses a positive coordinated antitumor effect along with reliable safety *in vitro*.

## Discussion

The current treatment options for ovarian cancer include traditional surgical treatment combined with chemotherapy, as well as targeted therapy and immunotherapy, which have recently emerged. Chemotherapy resistance is a major cause of both ineffective chemotherapy and tumor recurrence in patients. While cytotoxic chemotherapeutic drugs cause tumor tissue damage, tumor cell hypoxia, and apoptosis, they also trigger the release of cytokines and chemokines locally or systemically from mesenchymal cells in the TME, which are mainly macrophages, endothelial cells, and fibroblasts. Among them, macrophages, as the primary immune cells, are the main component of the TME,^29^ which has also been a hot research topic in recent years. Studies have indicated that infiltration of TAMs in the TME may increase after chemotherapy, which diminishes the chemotherapy drug-induced killing effect of tumor cells and creates chemoresistance.^7^ Previous studies in our laboratory also found that the proportion of M2-type macrophages in the ascites of ovarian cancer patients after conventional chemotherapy was higher than that before chemotherapy; in the mouse, ID8 ovarian cancer peritoneal animal model, paclitaxel triggered a significantly higher proportion of peritoneal TAMs than controls by inducing the release of phosphatidylserine molecules from apoptotic tumor cells. The possibility of combining therapy with small molecule inhibitors of macrophage-associated receptors to improve the therapeutic effect of tumors is the focus of this study.

CSF-1R exerts an important role in regulating the proliferation and differentiation of macrophages as well as a key factor in the phenotypic alteration of macrophages.^9^ In clinical studies on CSF-1R expression, it has been found that in various solid tumors, such as pancreatic and breast cancers, as well as hematologic malignancies, such as classical Hodgkin's lymphoma, up-regulation of CSF-1R expression leads to a regulatory imbalance of normal cells that promotes tumorigenesis and progression. In studies of breast cancer, normal breast tissue did not express CSF-1R, but it was up-regulated in 58% of breast cancer patients, and up to 85% of invasive breast cancer patients expressed CSF-1R.^30^ Additionally, data showed that high expression of CSF-1R was associated with lymph node metastasis.^31^ In our present research, the proportion of patients older than 50 years was 53.2%, cases of histological grade III accounted for 67.6%, plasmacytoma had the largest proportion among the histological types (45.9%), FIGO stages III/IV were mainly found (78.4%), and 80.2% of the patients had recurrence(s). From the above data, we found that the onset time of ovarian cancer is late, the pathology of ovarian cancer is mostly plasmacytosis, and the disease is mostly at an advanced stage when detected, with an extremely high recurrence rate. Furthermore, higher CSF-1R expression was found to be significantly associated with patient tumor size, lymph node metastasis, and FIGO stage in this study, consistent with the literature.^31^

CSF − 1R is a transmembrane tyrosine kinase receptor, and the binding of CSF-1 to CSF-1R on the cell surface exerts pro-tumor effects. The binding of CSF-1 to CSF-1R triggers a series of intracellular signaling cascade responses, and the phosphorylation of multiple autophosphorylation sites contained within the CSF-1R kinase region can activate the initiation of downstream signaling pathways, such as MAPK, PI3K, and PLCγ, thus initiating and regulating downstream regulators involved in cell proliferation, differentiation, and apoptosis.^32^ In addition, CSF1 binding to CSF-1R activates the autocrine pathway of CSF-1, which endows the tumor with invasive and metastatic properties. Espinosa et al found that the binding of CSF-1 to CSF-1R resulted in the production of several proangiogenic factors by tumor cells and accelerated the formation of tumor microvessels.^33^ Carlos et al found in peripheral T cell lymphoma, CSF-1 caused autophosphorylation of CSF-1R and activation of malignant T cells in an autocrine or paracrine manner, and inhibition of CSF-1R could reduce the growth of T cell lymphoma *in vitro*.^34^ Therefore, to understand whether inhibition of CSF-1R can play an anti-ovarian cancer role, we first selected the CSF- 1R small molecule inhibitor PLX3397 for *in vitro* experimental validation. *In vitro* experiments have demonstrated that PLX3397 can effectively inhibit the proliferation of various ovarian cancer cells by CCK8 assay (A2780, ID8), and when combined with the chemotherapeutic drug paclitaxel, the effect of inhibiting the proliferation of ovarian cancer cells is more obvious, and it enhanced the sensitivity of ovarian cancer cells to low concentrations of paclitaxel. PLX3397 promoted apoptosis in ovarian cancer cells as indicated by flow cytometry, and it promoted cell cycle arrest in the G1 phase as revealed by cell cycle assays. High expression of CSF-1R in ovarian cancer was speculated to be closely associated with the invasion and metastasis of malignant tumors.^35^ Persistent expression of CSF-1R apparently enhanced the invasiveness of ovarian cancer cells.^36^^,^^37^ CSF-1R facilitated the dissociation of the tumor cell adhesion molecule E-calmodulin from cytoskeletal proteins, which disrupted cell junctions and reduced the adhesion capacity, contributing to the ease of distant migration and proliferation of cancer cells away from their primary sites^37^. For this reason, we performed Transwell cell migration and invasion experiments to observe whether PLX3397 had an inhibitory effect on tumor metastasis to distant sites. The results revealed that the ability of ovarian cancer cells treated with PLX3397 to cross the stromal gel (simulating distant metastasis) was markedly reduced compared with that of the control cells, indicating that PLX3397 was able to reduce the potential of ovarian cancer cells to infiltrate and metastasize.

We further researched the antitumor effects and possible mechanisms of PLX3397 *in vivo*. First, we constructed an ID8 ovarian cancer chemotherapy model in mice to simulate intraperitoneal chemotherapy in clinical ovarian cancer patients. After 10 days of inoculation treatment of murine poorly differentiated plasmacytoid carcinoma ID8 cells, paclitaxel (15 mg/kg) was administered once intraperitoneally, and 72 h later, the cells were extracted from the peritoneal cavity of mice for flow cytometry analysis. As a result, the proportion of CSF-1R-positive M2 macrophages in the peritoneal cavity of paclitaxel-treated mice was found to be increased compared with that in other groups, which is consistent with a previous study in our laboratory. Therefore, we believe that CSF-1R can be used as a therapeutic target for ovarian cancer. We observed whether the CSF-1R inhibitor PLX3397 could enhance the antitumor effect of paclitaxel *in vivo* and control ascites formation and reduce intraperitoneal implant metastasis. In this experiment, we found that the effect of PLX3397 alone in reducing abdominal tumor nodules was slightly inferior to that of paclitaxel, but it was stronger than paclitaxel in reducing ascites production. When both drugs were combined, they significantly reduced the number of tumor nodules and the volume of malignant ascites in the abdominal cavity of tumor-bearing mice; at the same time, the survival period was significantly prolonged, and the drug effect was better than that of PLX3397 or paclitaxel alone. The results of the above-mentioned *in vivo* experiments showed that PLX3397 slowed tumor growth to some extent, but when combined with paclitaxel, the inhibition of tumor growth and ascites formation was stronger.

CSF-1R is an important factor that regulates macrophage proliferation, differentiation, and phenotypic alteration.^10^ Therefore, we detected macrophages in the peritoneal TME in each group of tumor-bearing mice by flow cytometry and found that PLX3397 alone and its combination therapy both significantly reduced the elevation of M2-type macrophages. We also found that there was a significant increase in M1 macrophages in the PLX3397 monotherapy group and combination therapy group, and the ratio value of M1/M2 was lower in the solvent control group and paclitaxel monotherapy group, while it was clearly increased in the PLX3397 monotherapy group and combination therapy group, which indicated that PLX3397 might enhance the antitumor effect of paclitaxel by modulating the phenotypic alterations of macrophages in the ascites TME. In addition, we analyzed other TME indicators (MDSCs, neutrophils, inflammatory monocytes, CD4^+^ T cells, and CD8^+^ T cells) in the peritoneal cavity of the tumor-bearing mice to understand the immune changes. The results showed that the TME in the ascites of paclitaxel-treated tumor-bearing mice was immunosuppressed, which might be the cause of paclitaxel chemoresistance. However, in the tumor-bearing mice treated with PLX3397 and paclitaxel, there was a positive immune modulation, and the proportion of M2 macrophages, MDSCs, neutrophils, and inflammatory monocytes in the ascites TME was reduced, while the CD8^+^ T cell proportion was increased, suggesting that the combination treatment not only reduced the suppression of natural immunity but also enhanced the function of the acquired immunity.

In conclusion, this study showed a close correlation between CSF-1R and the prognosis of ovarian cancer patients. PLX3397 enhanced the antitumor effect of paclitaxel both in the *in vivo* and *ex vivo* experiments without obvious drug-toxic side effects. The possible mechanism is to regulate the M2 macrophage reduction in the peritoneal cavity and tumor tissues, increase the numbers of antineoplastic M1 macrophages and T cells, and inhibit the cell proliferation and blood vessel formation of ovarian cancer. CSF-1R is expected to be a potential target in ovarian cancer treatment.

## Ethics declaration

The tumor specimens and clinical data of patients were provided by Shanghai Xinchao Biotechnology Co., Ltd. and were ethically approved. All animal experiments strictly follow the guidelines of the State Key Laboratory of Biotherapy Animal Care and Use Committee of Sichuan University.

## Author contributions

X. Wei and X. Zhao conceived and supervised the research, and designed the experiments. M. Yu, W. Hong, and Y. Yang developed the methodology of analysis. M. Yu, X. Hu, and Y. Yang helped with data collection and acquisition. M. Yu, Q. Li, and T. Lu analyzed the data. M. Yu and Y. Wu wrote the manuscript. All authors read and approved the final version of the manuscript.

## Conflict of interests

Xiawei Wei is the member of *Genes & Diseases* Editorial Board. To minimize bias, he/she was excluded from all editorial decision-making related to the acceptance of this article for publication. The remaining authors declare no conflict of interests.

## Funding

This work was supported by the National Science Foundation for Excellent Young Scholars (China) (No. 32122052), and the National Natural Science Foundation Regional Innovation and Development (China) (No. U19A2003).

## Figures and Tables

**Fig. S1(A) Flow cytometry data on induction of apoptosis in ovarian cancer cells by PLX3397 and paclitaxel in vitro. (B) PLX3397 inhibited the migration and invasion of ID8 ovarian cancer cells in vitro. PLX3397 combined with paclitaxel enhanced the migration and invasion of ID8 ovarian cancer cells in vitro. Data were presented as mean ± SD. n = 3. ∗P < 0.05, ∗∗P < 0.01, ∗∗∗P < 0.001, ∗∗∗∗P < 0.0001 (Student's t-test):**
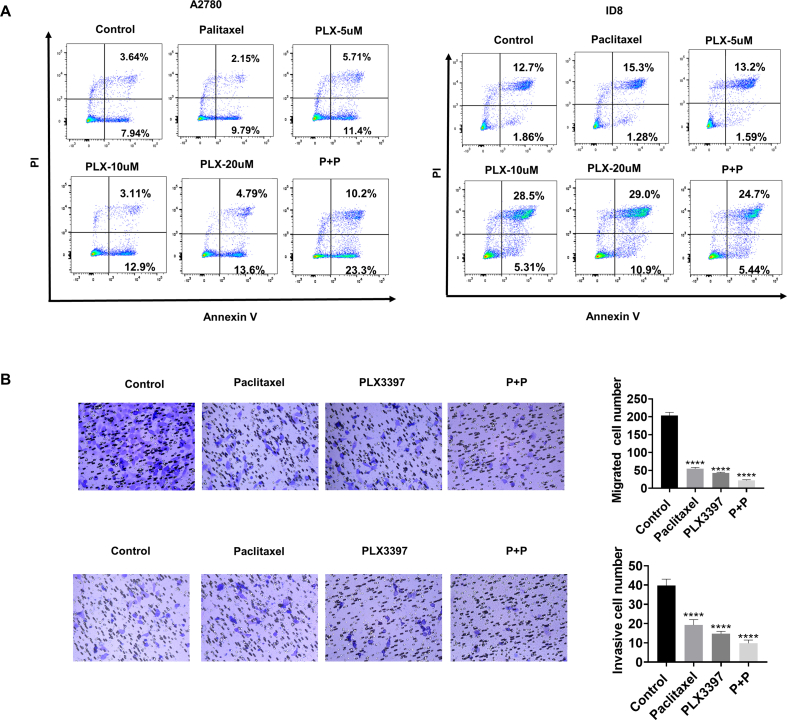


**Fig. S2Drug treatment did not affect CSF-1 and CSF-1R expression in ID8 cells in vitro, but PLX3397 could antagonize the paclitaxel-induced increase in CSF-1R phosphorylation levels to some extent. (A) The expression of CSF-1, CSF-1R, and p-CSF-1R in ID8 cells of different treatment groups was detected by western blot. GAPDH was used as a loading control (n = 3.) (B, C) Quantification results of western blot of CSF-1, CSF-1R, and p-CSF-1R. Data were presented as mean ± SD. n = 3. ∗P < 0.05, ∗∗P < 0.01, ∗∗∗P < 0.001, ∗∗∗∗P < 0.0001 (Student's t-test):**
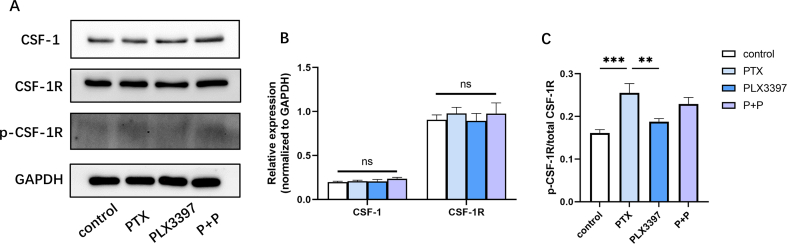


**Fig. S3PLX3397 inhibited neoangiogenesis and cell proliferation in ovarian cancer cells after paclitaxel chemotherapy. (A–C) The expression of CD31 (A), VEGF (B), and Ki67 (C) in ovarian cancer tissues in each group of mice was measured by using immunofluorescence. DAPI-stained nuclei were in blue. Scale bars = 200 μm:**
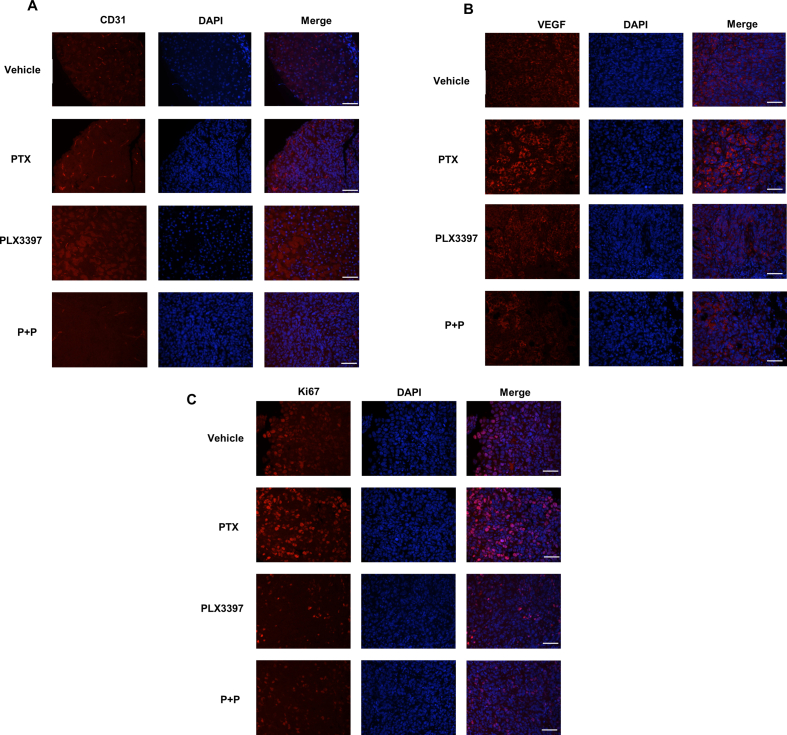


**Fig. S4PLX3397 prevented macrophage M2 polarization in vitro by inhibiting AKT phosphorylation downstream of CSF-1R. (A) The expression of Arg-1, p-AKT, and AKT in macrophages was detected by western blot. GAPDH was used as a loading control. n = 3. (B, C) Quantification results of western blot of Arg-1, p-AKT, and AKT. Data were presented as mean ± SD. n = 3. ∗P < 0.05, ∗∗P < 0.01, ∗∗∗P < 0.001, ∗∗∗∗P < 0.0001 (Student's t-test). (D, E) Flow cytometry was used to analyze the proportion (D) and count (E) of macrophages:**